# MRI Surveillance and Breast Cancer Mortality in Women With *BRCA1* and *BRCA2* Sequence Variations

**DOI:** 10.1001/jamaoncol.2023.6944

**Published:** 2024-02-29

**Authors:** Jan Lubinski, Joanne Kotsopoulos, Pal Moller, Tuya Pal, Andrea Eisen, Larissa Peck, Beth Y. Karlan, Amber Aeilts, Charis Eng, Louise Bordeleau, William D. Foulkes, Nadine Tung, Fergus J. Couch, Robert Fruscio, Teresa Ramon y Cajal, Christian F. Singer, Susan L. Neuhausen, Dana Zakalik, Cezary Cybulski, Jacek Gronwald, Tomasz Huzarski, Klaudia Stempa, Jeffrey Dungan, Carey Cullinane, Olufunmilayo I. Olopade, Kelly Metcalfe, Ping Sun, Steven A. Narod

**Affiliations:** 1 International Hereditary Cancer Center, Department of Genetics and Pathology, Pomeranian Medical University, Szczecin, Poland; 2 Women’s College Research Institute, University of Toronto, Toronto, Ontario, Canada; 3 Dalla Lana School of Public Health, University of Toronto, Toronto, Ontario, Canada; 4 Institute of Cancer Research, Department of Tumour Biology, The Norwegian Radium Hospital, Oslo University Hospital, Oslo, Norway; 5 Vanderbilt-Ingram Cancer Center, Vanderbilt University Medical Center, Nashville, Tennessee; 6 Department of Medical Oncology, Odette Cancer Center, University of Toronto, Toronto, Ontario, Canada; 7 Bhalwani Familial Cancer Clinic, Princess Margaret Cancer Centre, Toronto, Ontario, Canada; 8 Department of Obstetrics and Gynecology, David Geffen School of Medicine at UCLA, University of California, Los Angeles, Los Angeles, California; 9 Comprehensive Cancer Center, Division of Human Genetics, The Ohio State University Medical Center, Columbus; 10 Genomic Medicine Institute, Center for Personalized Genetic Healthcare, Cleveland Clinic, Cleveland, Ohio; 11 Department of Oncology, Juravinski Cancer Centre, Hamilton, Ontario, Canada; 12 McGill Program in Cancer Genetics, Department of Oncology, McGill University, Montreal, Quebec, Canada; 13 Cancer Risk and Prevention Program, Beth Israel Deaconess Medical Center, Boston, Massachusetts; 14 Division of Experimental Pathology and Laboratory Medicine, Department of Laboratory Medicine and Pathology, Mayo Clinic, Rochester, Minnesota; 15 Department of Medicine and Surgery, University of Milano-Bicocca, IRCCS San Gerardo, Monza, Italy; 16 Hospital de la Santa Creu i Sant Pau, Barcelona, Spain; 17 Department of Obstetrics and Gynecology and Comprehensive Cancer Center, Medical University of Vienna, Vienna, Austria; 18 Division of Biomarkers of Early Detection and Prevention, City of Hope, Duarte, California; 19 Cancer Genetics Program, Beaumont Hospital, Royal Oak, Michigan; 20 Northwestern Medical Group, Chicago, Illinois; 21 Long Beach Memorial Center, Long Beach, California; 22 Department of Medicine and Human Genetics, University of Chicago, Chicago, Illinois; 23 Bloomberg School of Nursing, University of Toronto, Toronto, Ontario, Canada

## Abstract

**Importance:**

Magnetic resonance imaging (MRI) surveillance is offered to women with a pathogenic variant in the *BRCA1* or *BRCA2* gene who face a high lifetime risk of breast cancer. Surveillance with MRI is effective in downstaging breast cancers, but the association of MRI surveillance with mortality risk has not been well defined.

**Objective:**

To compare breast cancer mortality rates in women with a *BRCA1* or *BRCA2* sequence variation who entered an MRI surveillance program with those who did not.

**Design, Setting, and Participants:**

Women with a *BRCA1* or *BRCA2* sequence variation were identified from 59 participating centers in 11 countries. Participants completed a baseline questionnaire between 1995 and 2015 and a follow-up questionnaire every 2 years to document screening histories, incident cancers, and vital status. Women who had breast cancer, a screening MRI examination, or bilateral mastectomy prior to enrollment were excluded. Participants were followed up from age 30 years (or the date of the baseline questionnaire, whichever was later) until age 75 years, the last follow-up, or death from breast cancer. Data were analyzed from January 1 to July 31, 2023.

**Exposures:**

Entrance into an MRI surveillance program.

**Main Outcomes and Measures:**

Cox proportional hazards modeling was used to estimate the hazard ratios (HRs) and 95% CIs for breast cancer mortality associated with MRI surveillance compared with no MRI surveillance using a time-dependent analysis.

**Results:**

A total of 2488 women (mean [range] age at study entry 41.2 [30-69] years), with a sequence variation in the *BRCA1* (n = 2004) or *BRCA2* (n = 484) genes were included in the analysis. Of these participants, 1756 (70.6%) had at least 1 screening MRI examination and 732 women (29.4%) did not. After a mean follow-up of 9.2 years, 344 women (13.8%) developed breast cancer and 35 women (1.4%) died of breast cancer. The age-adjusted HRs for breast cancer mortality associated with entering an MRI surveillance program were 0.20 (95% CI, 0.10-0.43; *P* < .001) for women with *BRCA1* sequence variations and 0.87 (95% CI, 0.10-17.25; *P* = .93) for women with *BRCA2* sequence variations.

**Conclusion and Relevance:**

Results of this cohort study suggest that among women with a *BRCA1* sequence variation, MRI surveillance was associated with a significant reduction in breast cancer mortality compared with no MRI surveillance. Further studies of women with *BRCA2* sequence variations are needed to ascertain these women obtain the same benefits associated with MRI surveillance.

## Key Points

**Question:**

What is the breast cancer mortality risk of women with a *BRCA1* or *BRCA2* sequence variation after entering a magnetic resonance imaging (MRI) surveillance program?

**Findings:**

This cohort study included 1442 women with *BRCA1* and 314 with *BRCA2* sequence variations who underwent a mean of 4.7 screening MRI examinations. At 20 years, the risk of breast cancer mortality was 3.2% in the MRI surveillance group compared with 14.9% for women who did not undergo MRI surveillance.

**Meaning:**

Results of this study suggest that among women with a *BRCA1* sequence variation, MRI surveillance is associated with reduced breast cancer mortality risk.

## Teaser

This cohort study examines the association between magnetic resonance imaging (MRI) surveillance and the risk of breast cancer mortality in women with *BRCA1* or *BRCA2* sequence variations.

## Introduction

Women with a pathogenic sequence variation in the *BRCA1* or *BRCA2* gene face a lifetime risk of breast cancer of 70%.^1^ Approximately one-third of these women undergo risk-reducing mastectomy, but the majority opt for intensified surveillance.^2^ In North America, women with a *BRCA1* or *BRCA2* sequence variation are recommended to have annual screening with magnetic resonance imaging (MRI) from age 25 or 30 years to age 70 years,^3,4^ and it is important to measure the effectiveness of MRI surveillance in terms of mortality reduction. Given that lead time bias and overdetection of cancer may affect survival rates, there is a need to compare mortality rates among women with *BRCA1* or *BRCA2* sequence variations who undergo MRI surveillance with those who do not. Here we report on long-term breast cancer incidence and cumulative breast cancer mortality of a cohort of women with a *BRCA1* or *BRCA2* sequence variation who did or did not undergo MRI surveillance. We excluded women diagnosed with breast cancer or who had a screening MRI examination or bilateral mastectomy prior to study enrollment to focus on the association between MRI surveillance and mortality risk among women who face a choice between bilateral preventive mastectomy and MRI surveillance.

## Methods

### Study Population

Eligible participants were identified for this cohort study from a longitudinal study of 17 940 women with *BRCA1* or *BRCA2* sequence variations that began in 1995 and now includes 59 participating centers in 11 countries (US, Canada, Poland, Norway, Israel, Italy, Austria, the Netherlands, France, Spain, and the Bahamas). Sequence variations were detected using a range of techniques; all abnormal nucleotide sequences were confirmed by direct DNA sequencing. All participants provided written informed consent and the institutional ethics committee of each participating center approved the study.

### Data Collection

Participants completed a baseline questionnaire at enrollment. A follow-up questionnaire was administered every 2 years thereafter to update exposures and to ascertain incident cancers and deaths. Questionnaires were mailed to the participants, administered over the telephone by a genetic counselor or research assistant, or completed online by the participant. The questionnaires requested information regarding surgery (eg, bilateral oophorectomy, hysterectomy, mastectomy, tubal ligation) and hormone use (eg, hormone replacement therapy, oral contraceptives). Women were asked if they had participated in an MRI surveillance program and, if so, the date of their first MRI examination, the date of their most recent MRI examination, and the total number of MRI examinations. For those who developed breast cancer, we asked for the date of the most recent MRI examination prior to or at the time of breast cancer diagnosis. For the purpose of this study, we considered any MRI examination done in any year prior to the year of breast cancer diagnosis to be a screening MRI examination. If the year of first MRI examination and the year of breast cancer diagnosis were the same, we sought the indication for MRI (diagnostic or screening).

### Incident Cancer Diagnoses, Vital Status, and Cause of Death

Women who developed breast cancer were asked if the cancer was in situ or invasive and how it was detected (eg, self-examination, MRI, physical examination, mammography, ultrasonography, or other means [including unknown]). Pathology reports and medical records were requested for all women who reported incident breast cancer and their hormone receptor status, tumor size, and nodal status were recorded. Women were asked if they received chemotherapy or tamoxifen for treatment and the type of surgery they underwent. The cause and date of death were requested from the collaborating investigator and were determined by review of patient medical records, by correspondence with the treating physician, or by contact with the participant’s next of kin. In Ontario, Canada, the date and cause of death were determined by record linkage to the Ontario Cancer Registry. In Poland, the date of death was determined by record linkage to the Polish Vital Statistics Database. For 7 participants, the cause of death was listed as breast cancer, but no incident cancer was reported prior to the death. In these cases, we considered the date of breast cancer diagnosis to be 1 year prior to the date of death.

### Inclusion and Exclusion Criteria

The study was restricted to women born between 1940 and 1990. Women were excluded if they had been diagnosed with any cancer (except for thyroid or nonmelanoma skin cancer) in the same year or prior to the completion of the baseline questionnaire or if they did not complete at least 1 follow-up questionnaire. Women were also excluded if they had a bilateral mastectomy prior to baseline or had a screening MRI examination prior to baseline. Eight women who developed breast cancer before age 30 years were excluded. Women were also excluded if they: (1) completed the baseline questionnaire after age 70 years; (2) completed the most recent follow-up questionnaire before age 35 years; (3) completed the most recent follow-up questionnaire before 2000; or (4) were missing data on key variables (eg, date of birth, MRI use, number of MRI examinations).

### Statistical Analysis

The primary end point was breast cancer–specific survival. All participants were followed up from the date of the baseline questionnaire or age 30 years (whichever came last) until death from any cause, bilateral risk-reducing mastectomy, age 75 years, or the date of completion of the last follow-up questionnaire. In all, 1756 of 2448 women had 1 or more screening MRI examinations after the date of the baseline questionnaire (mean time elapsed from baseline to first screening MRI examination, 3.6 years). These women were considered to be unexposed to MRI surveillance from the baseline date to the date of the first screening MRI examination, and these person-years were accrued in the no MRI surveillance arm. Participants were then followed up in the MRI surveillance group after the date of the first screening MRI examination until death from any cause, bilateral risk-reducing mastectomy, age 75 years, or the date of completion of the last follow-up questionnaire. We estimated the extent of risk reduction for breast cancer mortality associated with MRI surveillance using a cause-specific Cox proportional hazards model with the date of first screening MRI examination as a time-dependent variable and death from other causes as a competing risk. The hazard ratios (HRs) were adjusted for age at study entry, gene variant (*BRCA1* or *BRCA2*), country of residence (Canada, US, Poland, or other), and oophorectomy status (time dependent). The analysis was left-censored to age 30 years.

We estimated the cumulative risk of breast cancer mortality in women exposed and unexposed to MRI surveillance, with death from breast cancer as the end point and deaths from other causes considered as a competing risk. Women in the MRI surveillance group were followed up from the date of first screening MRI examination and women in the no MRI surveillance group were followed up from baseline. We also conducted a secondary analysis in which we compared breast cancer mortality among women who did and did not have a screening MRI examination by calendar age, from ages 30 to 75 years, with death from other causes as a competing risk.

We estimated the 20-year cumulative incidence of breast cancer for women exposed to and unexposed to MRI surveillance. To calculate the 20-year cumulative breast cancer incidence for women unexposed to MRI surveillance, they were followed up from baseline to breast cancer diagnosis (censored at first screening MRI examination, bilateral mastectomy, death, or date of last follow-up). To calculate the 20-year cumulative breast cancer incidence after the first screening MRI examination, we followed up participants from the date of the first screening MRI examination to the date of breast cancer diagnosis, bilateral mastectomy, death, or date of last follow-up. The cumulative incidence curves and corresponding HRs were generated using the Cox proportional hazards model adjusted for age.

We compared the characteristics of the breast cancers diagnosed in women who were or were not exposed to MRI surveillance (invasive vs noninvasive cancer, tumor size, and lymph nodes involvement (yes or no). We compared the characteristics of breast cancer in the women in the MRI surveillance group according to the means of detection (MRI vs other). We described the clinical histories of the women who died of breast cancer after entering an MRI surveillance program. Data were analyzed between January 1 and July 31, 2023, using SAS, version 9.4 (SAS Institute Inc). *P* values were based on 2-sided tests and statistical significance was set at *P* < .05.

## Results

The study cohort consisted of 2488 women with a sequence variation in the *BRCA1* (n = 2004) or *BRCA2* (n = 484) gene. The mean (range) age at study entry was 41.2 (30 to 69) years; 25 women were Asian (1.0%), 5 were Black (0.2%), and 2421 were White (97.3%), and 37 individuals were of other race or ethnicity (1.5%). A total of 1756 women (71%) in the cohort had at least 1 screening MRI examination, either in the baseline year (n = 505) or thereafter (n = 1251). For those who had the first screening MRI examination after baseline, the mean (range) time elapsed from baseline to the first screening MRI examination was 3.6 (1-19) years. The mean (range) age at the first screening MRI examination was 43.2 (30-69) years and mean (range) age at end of follow-up was 50.6 (35-75) years. Of the 1756 women in the MRI surveillance group, the mean (range) number of screening MRI examinations was 4.7 (1-16). Of the 1365 women who had 2 or more screening MRI examinations, the mean (range) interval between MRI examinations was 0.95 (0.1 to 6.0) years. Of the 1756 women in the MRI surveillance group, 245 (14.0%) underwent a preventive mastectomy at a later date compared with 119 (16.3%) of the 732 women in the no MRI surveillance group. Breast cancer was detected at the time of mastectomy in 11 patients; however, none of the 364 women who underwent mastectomy died of breast cancer during a mean (range) follow-up of 5.3 (0.1-21) years after diagnosis. Of the 732 women in the no MRI surveillance group, 636 (86.7%) reported having had at least 1 mammogram. Additionally, women in the MRI surveillance group were more likely to have had a preventive oophorectomy than those in the no MRI surveillance group (66% vs 46%). Table 1 shows the characteristics of women who underwent MRI surveillance and those who did not.

**Table 1.** Characteristics of Participants With *BRCA1* or *BRCA2* Sequence Variations Stratified by Magnetic Resonance Imaging (MRI) Surveillance Status

Variable	No. (%)	
	No MRI surveillance (n = 732)	MRI surveillance (n = 1756)
Year of birth (range)	1961 (1940-1984)	1964 (1940-1985)
Year of baseline (range)	2003 (1995-2015)	2005 (1995-2015)
Age at study entry, mean (range), y	41.9 (20-68)	40.7 (19-69)
Age at start of follow-up, mean (range), y	42.2 (30-69)	43.2 (30-69)
Age at first MRI examination, mean (range), y	NA	43.2 (30-69)
Year of first MRI examination (range)	NA	2007 (1997-2018)
Age at last follow-up, mean (range), y	49.5 (35-75)	50.6 (35-75)
Year of last follow-up (range)	2011 (2000-2021)	2015 (2001-2022)
No. of years of follow-up from baseline, mean (range)	7.5 (0.1-23.6)	9.8 (0.1-24.5)
No. of years of follow-up from first screening MRI examination, mean (range)	NA	7.8 (0.3-24.2)
Screening MRI examinations, mean (range)	NA	4.7 (1-16)
Sequence variation		
* BRCA1*	562 (76.8)	1442 (83.1)
* BRCA2*	170 (23.2)	314 (17.9)
Bilateral mastectomy during follow-up		
No	613 (83.7)	1511 (86.1)
Yes	119 (16.3)	245 (14.0)
Oophorectomy		
No	399 (54.5)	601 (34.2)
Yes	333 (45.5)	1155 (65.8)
Tamoxifen or raloxifene use		
No	660 (94.2)	1556 (93.6)
Yes	42 (5.8)	107 (6.4)
Missing data	30	93
Ever underwent mammography		
No	97 (12.6)	212 (12.1)
Yes	636 (86.7)	1544 (87.9)
Breast cancer type		
All types	103 (14.1)	241 (13.7)
Invasive	79 (10.8)	205 (11.7)
DCIS	17 (2.3)	33 (1.9)
Missing data	7	3
Other cancers^a^	49 (6.7)	139 (7.9)
Country of residence		
Canada	178 (24.3)	448 (25.5)
Poland	254 (34.7)	871 (49.6)
US	186 (25.4)	204 (11.6)
Norway	10 (1.4)	164 (9.3)
Other^b^	104 (14.2)	69 (3.9)
Vital status at end of follow-up		
Dead	47 (6.4)	45 (2.6)
Died of breast cancer	21 (2.8)	14 (0.8)
Died of ovarian or peritoneal cancer	13 (1.8)	17 (1.0)
Died of other cause	9 (1.2)	12 (0.7)
Cause of death unknown	4 (0.5)	2 (0.1)

Abbreviations: DCIS, ductal carcinoma in situ; NA, not applicable.

^a^ Includes brain, colon, lung, endometrial, kidney, pancreatic, liver, and thyroid cancers (eTable 1 in Supplement 1).

^b^ Includes the Netherlands, Austria, Italy, Spain, France, China, and Israel.

The 2488 women were followed up for up to 24 years (mean [range], 9.2 [0.1 to 24.5] years) from baseline for incident breast cancers and death. There were 344 incident breast cancers (13.8%) reported in the cohort (284 invasive, 50 ductal carcinoma in situ, and 10 missing data) and 92 deaths (35 from breast cancer [1.4%], 51 from other causes, and 6 missing data) (eTable 1 in Supplement 1). Among the 1756 women who underwent MRI surveillance, there were 241 breast cancers and 14 deaths from breast cancer. In 5 of these 14 women, breast cancer was diagnosed on the first screening MRI examination; in 6 of these women, more than 1 year had elapsed since their preceding MRI examination. Among the 732 women who did not undergo MRI surveillance, there were 103 breast cancers and 21 deaths from breast cancer. After adjustment for age, *BRCA* sequence variation, country of residence, and oophorectomy status, the HR for breast cancer mortality associated with entering the MRI surveillance program (time dependent) was 0.23 (95% CI, 0.11-0.48; *P* = .001) (Table 2). The HR was 0.20 (95% CI, 0.10-0.43; *P* < .001) for women with the *BRCA1* sequence variation and 0.87 (95% CI, 0.10-17.25; *P* = .93) for women with the *BRCA2* sequence variation. Additionally, the HR for breast cancer mortality for MRI-exposed women, compared with mammography-exposed women, was 0.27 (95% CI, 0.12-0.58; *P* < .001). There were 92 deaths in the entire cohort before age 75 years (eTable 1 in Supplement 1). The HR for all-cause mortality associated with MRI surveillance was 0.42 (95% CI, 0.26-0.66; *P* = .001). There were 188 cancers other than breast cancer reported in the cohort (eTable 2 in Supplement 1).

**Table 2.** Breast Cancer–Specific Mortality and All-Cause Mortality Stratified by Magnetic Resonance Imaging (MRI) Surveillance Status^a^

Events	No./total No. of deaths (%)	HR (95% CI)	*P* value
All deaths			
Breast cancer death^b^			
No MRI surveillance	21/732 (2.9)	1 [Reference]	NA
MRI surveillance	14/1756 (0.8)	0.23 (0.11-0.48)	<.001
*BRCA1* sequence variation			
Breast cancer death			
No MRI surveillance	18/562 (3.2)	1 [Reference]	NA
MRI surveillance	12/1442 (0.8)	0.20 (0.10-0.43)	<.001
*BRCA2* sequence variation			
Breast cancer death			
No MRI surveillance	3/170 (1.8)	1 [Reference]	NA
MRI surveillance	2/317 (0.6)	0.87 (0.10-17.25)	.93
All-cause mortality			
No MRI surveillance	47/732 (6.4)	1 [Reference]	NA
MRI surveillance	45/1756 (2.6)	0.42 (0.26-0.66)	.001

Abbreviations: HR, hazard ratio; NA, not applicable.

^a^ Analyses adjusted for country, *BRCA* sequence variation, age at baseline, and oophorectomy status. In calculating HRs, screening MRI was considered a time-dependent variable and death from other causes was a competing risk.

^b^ For all participants, for breast cancer–specific death, follow-up started from age at baseline, left-truncated at 30 years. Participants in the MRI surveillance group were transferred from the no MRI surveillance group to the MRI surveillance group at the date of the first screening MRI examination.

Among the 241 women who underwent MRI surveillance and were diagnosed with breast cancer, 77 cancers were palpable, 148 were detected by screening, and 15 were detected by another method (eTable 3 in Supplement 1). Of the 148 cancers that were detected via screening, 106 were detected by MRI. Among the 103 women who did not undergo MRI surveillance and developed breast cancer, 59 cancers were palpable, 28 were detected by screening, and 16 were detected by another method.

**Figure 1.**
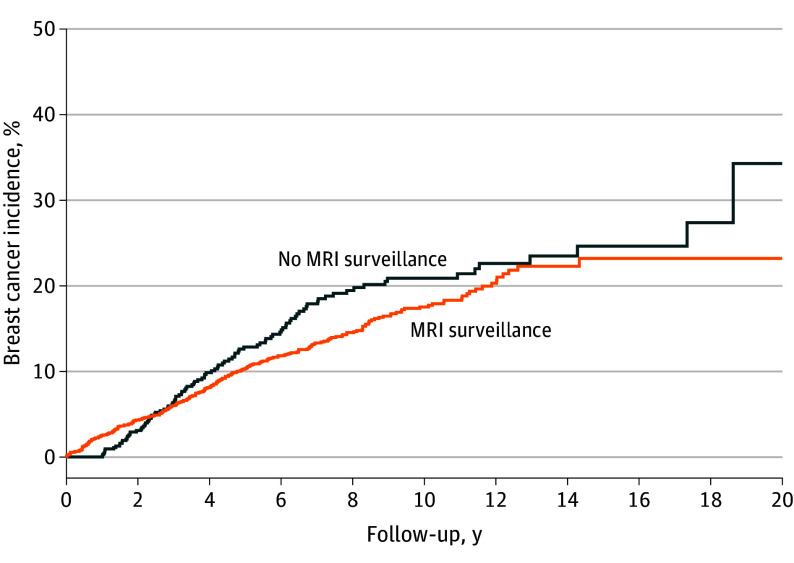
Incidence of Breast Cancer Over 20 Years of Follow-Up in Women with *BRCA1* or *BRCA2* Sequence Variations Stratified by Magnetic Resonance Imaging (MRI) Surveillance Status The follow-up for the MRI surveillance group started at the date of first screening MRI examination; follow-up for the no MRI surveillance group started at baseline. If age at baseline was 30 years or younger, follow-up started at age 30 years.

The cumulative incidence of breast cancer in the 2 groups was similar (Figure 1). The age-adjusted HR for breast cancer in the MRI surveillance group vs no MRI surveillance group was 1.12 (95% CI, 0.88-1.43; *P* = .35). The HR for invasive breast cancer with MRI surveillance vs no MRI surveillance was 1.19 (95% CI, 0.91-1.56; *P* = .20). The HR for ductal carcinoma in situ in the MRI surveillance group vs the no MRI surveillance group was 0.94 (95% CI, 0.51-1.73; *P* = .85).

**Figure 2.**
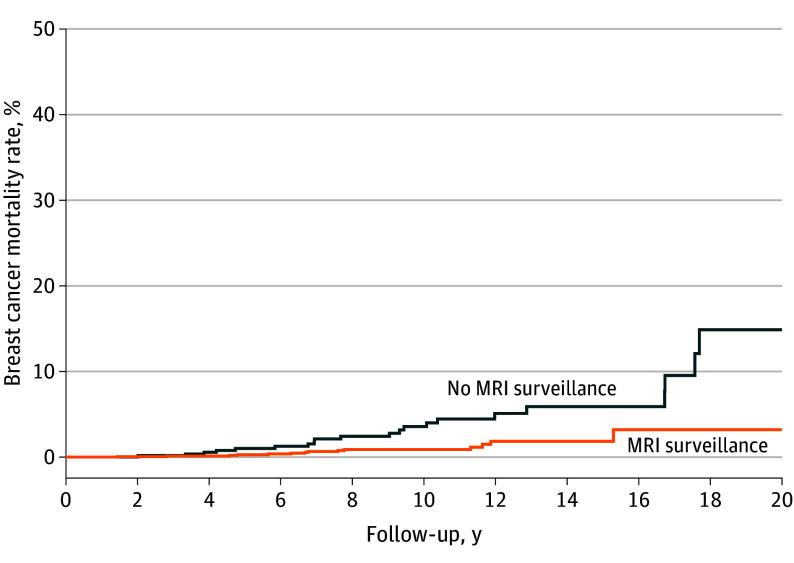
Breast Cancer Mortality Over 20 Years in Women with *BRCA1* or *BRCA2* Sequence Variations, By Magnetic Resonance Imaging (MRI) Surveillance Status The follow-up for the MRI surveillance group started at the date of first screening MRI examination; follow-up for the no MRI surveillance group started at baseline. If age at baseline was 30 years or younger, follow-up started at age 30 years.

The annual breast cancer mortality rates by age (in 10-year intervals) according to MRI surveillance status are presented in Table 3. Among the women in the no MRI surveillance group, the cumulative risk of breast cancer mortality at 20 years from baseline was 14.9% (annual risk, 0.4%) (Figure 2). After the first screening MRI examination in the MRI surveillance group, the cumulative risk of breast cancer mortality was 3.2% at 20 years (annual risk, 0.1%). If we restrict follow-up to those whose first screening MRI examination results were negative for breast cancer, the cumulative risk of breast cancer mortality at 20 years from the date of the first screening MRI examination was 2.9% (annual risk, 0.1%). We also estimated the cumulative breast cancer mortality rate from ages 30 to 75 years for the 2 groups. The cumulative risk of breast cancer mortality was 20.5% to age 75 years for those who did not undergo MRI surveillance and 5.5% for those who underwent MRI surveillance (*P* < .001).

**Table 3.** Annual Breast Cancer Mortality Rates for Women With *BRCA1* or *BRCA2* Sequence Variations by Magnetic Resonance Imaging (MRI) Surveillance Status

Age group, y^a^	No MRI surveillance (n = 732)			After first screening MRI (n = 1756)		
	Person-years	No. of deaths from breast cancer	Annual mortality rate, %	Person-years	No. of deaths from breast cancer	Annual mortality rate, %
35 to <45	1786.72	8	0.45	4520.56	3	0.07
45 to <55	1739.53	5	0.29	4167.75	5	0.12
55 to <65	1454.51	6	0.41	3648.35	4	0.11
65-<75	494.92	2	0.40	1633.32	2	0.12
All	5299.82	21	0.40	13 520.07	14	0.10

^a^ For each age group, the annual mortality rate is calculated as total events divided by the person-years.

For the 205 women (11.7%) who had invasive cancer diagnosed in the MRI surveillance group, 10-year survival was 93.8%. For the 79 women (10.8%) who had invasive cancer diagnosed in the no MRI surveillance group, 10-year survival was 86.7% (*P* < .01). The characteristics of the breast cancers in the 2 groups are presented in eTable 4 in Supplement 1. There were 14 women in the MRI surveillance group who died of breast cancer (eTable 5 in Supplement 1). In the entire cohort, 364 women underwent bilateral preventive mastectomy during follow-up; there were no deaths from breast cancer in this subgroup.

## Discussion

In this cohort study of 2488 women with a *BRCA1* or *BRCA2* sequence variation, MRI surveillance was associated with reduced mortality in women with a *BRCA1* sequence variation compared with those not undergoing MRI surveillance. At 20 years, the cumulative incidence of breast cancer in the 2 groups was similar. However, the number of breast cancer deaths in the *BRCA2* cohort was small (n = 5) and the confidence limits were wide.

To our knowledge, this is the largest prospective study of MRI surveillance and mortality in women with a *BRCA1* or *BRCA2* sequence variation. We restricted the period of observation to between ages 30 and 70 years because this is the age range at which screening MRI is recommended.^3^ The study was observational and was not a direct comparison of results with MRI vs mammography; however, the majority of the women in the no MRI surveillance group (86.7%) had at least 1 screening mammogram and the HR for breast cancer mortality for MRI-exposed women, compared with mammography-exposed women, was 0.27 (95% CI, 0.12-0.58; *P* < .001).

Several studies have reported that the breast cancers detected by screening MRI are smaller and more likely to be node negative than those detected by mammography (ie, downstaging).^5,6,7,8,9,10,11,12^ In the Familial MRI Screening (FaMRIsc) trial^11^ from the Netherlands, investigators randomly assigned 1355 women with a family history of breast cancer to MRI or mammography. After 7 years, there were more invasive cancers detected in the MRI group than in the mammography group (24 vs 8), but the number of node-positive cancers was similar in the 2 groups and there were too few deaths to compare mortality rates. It is not clear if the results from studies of women without the *BRCA* sequence variation can be extrapolated to the population of women with *BRCA* sequence variations. Tumor stage at diagnosis and survival rates are important factors of success, but ultimately it is necessary to show that the intervention is associated with a decline in breast cancer mortality. When examining these questions, it is important that the exposed and unexposed groups are as similar to each other as possible. In this study, women in the MRI surveillance group were more likely to have had a preventive oophorectomy than those in the no MRI surveillance group (66% vs 46%), but oophorectomy status was adjusted for in data analysis.

In the current study, the 20-year mortality rate from breast cancer after the first screening MRI examination was 3.2%. In a recent cohort study from Ontario, Canada, among 489 women with a *BRCA1* or *BRCA2* sequence variation, the 20-year mortality from breast cancer after the first screening MRI examination was 2.0%.^13^ The difference between these 2 rates is small and may be due to chance. Moreover, the Ontario program is an organized screening program with quality control measures and automatic recall of enrolled women. Our international cohort study presents the experience of 2488 women attending 59 different screening MRI clinics in 11 countries.

In the MRI Screening (MRISC) study,^10^ Dutch investigators compared MRI surveillance with risk-reducing mastectomy in 2857 women with *BRCA* sequence variations. After a median follow-up of 10 years, among the women with *BRCA* sequence variations, there were 21 breast cancer deaths among 1729 women in the MRI surveillance cohort (1.2%) and 1 death among 1128 women in the mastectomy cohort (0.1%). Many women who enter an MRI surveillance program will ultimately choose to undergo risk-reducing mastectomy to avoid the inconvenience of annual screening as well as to reduce anxiety and the possibility of a breast cancer diagnosis and the need for treatment. In our study, a total of 364 participants opted for a mastectomy during follow-up. Breast cancer was detected at the time of mastectomy in 11 patients; however, none of the 364 women who underwent mastectomy died of breast cancer.

In the present study, 14 women died of breast cancer in the MRI surveillance group (eTable 5 in Supplement 1). For 5 of these participants, breast cancer was diagnosed on the first MRI screening (prevalent cases) and for 6 women, more than 1 year had elapsed since their preceding MRI. These data emphasize the importance of starting screening MRI examinations before age 35 years and adhering to an annual screening schedule.

### Limitations

There are several limitations to our study. Participants with breast cancer were followed up for a mean (range) of 5.3 (0.1-21) years after diagnosis. Overall, the women in the cohort were followed up until age 50 years; ideally, we should follow up all women until age 75 years to establish the lifetime risks of breast cancer. The screening MRI examinations were carried out in several countries according to local protocols and image interpretation was not centralized. Most participants were White and there were too few women of other races or ethnicities to compare effectiveness in different racial and ethnic groups. The years of screening MRI examination ranged from 1997 to 2018 and may not reflect current protocols. Similarly, we were not able to compare screening MRI with modern mammography protocols, such as tomosynthesis.

## Conclusion

This cohort study supports the recommendation^3^ that women with *BRCA1* sequence variations aged 30 years or older should be offered MRI surveillance. We observed an 80% reduction in breast cancer mortality for women with *BRCA1* sequence variations after they entered an MRI surveillance program. Further follow-up in women with *BRCA2* sequence variations is needed to ascertain whether these patients obtain the same benefits associated with MRI surveillance.

## Notes

Supplement 1. **eTable 1.** Causes of Death for the 92 Participants Who Died **eTable 2.** Other Cancers Diagnosed in the Cohort **eTable 3.** Means of Detection of Breast Cancer **eTable 4.** Characteristics of Invasive Breast Cancers in the No MRI Surveillance Group and the MRI Surveillance Group **eTable 5.** Characteristics of the 14 Women Who Died of Invasive Breast Cancer in the MRI Surveillance Group

Supplement 2.
Nonauthor Collaborators

Supplement 3.
Data Sharing Statement
