# Supplementary material for: MRI Surveillance and Breast Cancer Mortality in Women With BRCA1 and BRCA2 Sequence Variations
Source: JAMA Oncol. 2024 Feb 29;10(4):493–9. doi: 10.1001/jamaoncol.2023.6944 (PMC10905376; doi:10.1001/jamaoncol.2023.6944)
Supplement: Supplement 1. — eTable 1. Causes of Death for the 92 Participants Who Died eTable 2. Other Cancers Diagnosed in the Cohort eTable 3. Means of Detection of Breast Cancer eTable 4. Characteristics of Invasive Breast Cancers in the No MRI Surveillance Group and the MRI Surveillance Group eTable 5. Characteristics of the 14 Women Who Died of Invasive Breast Cancer in the MRI Surveillance Group [file jamaoncol-e236944-s001.pdf]

Supplemental Online Content

Lubinski J, Kotsopoulos J, Moller P, et al; for the Hereditary Breast Cancer Clinical Study Group. MRI surveillance and breast cancer mortality in women with *BRCA1* and *BRCA2* sequence variations. *JAMA Oncol.* Published online February 29, 2024. doi:10.1001/jamaoncol.2023.6944

eTable 1. Causes of Death for the 92 Participants Who Died

eTable 2. Other Cancers Diagnosed in the Cohort

eTable 3. Means of Detection of Breast Cancer

eTable 4. Characteristics of Invasive Breast Cancers in the No MRI Surveillance Group and the MRI Surveillance Group

eTable 5. Characteristics of the 14 Women Who Died of Invasive Breast Cancer in the MRI Surveillance Group

This supplemental material has been provided by the authors to give readers additional information about their work.

© 2024 Lubinski J et al. *JAMA Oncology*

eTable 1. Causes of Death for the 92 Participants Who Died

Cause of death	Frequency
Breast cancer	35
Ovarian/peritoneal cancer	30
Brain tumour	2
Colon cancer	2
Heart/Circulatory Failure	2
Lung cancer	2
Thrombosis	2
Accident	1
Amyloidosis	1
Brain Aneurysm	1
Diabetes	1
Endometrial cancer	1
Kidney cancer	1
Leukemia	1
Liver cancer	1
Pancreas cancer	1
Amyotrophic lateral sclerosis	1
Thyroid cancer	1
Death Cause Unknown	6
Total	92

eTable 2. Other Cancers Diagnosed in the Cohort

Cancer	Frequency
Ovarian	85
Fallopian	14
Peritoneal	12
Melanoma	11
Pancreatic	11
Endometrial	8
Colon	7
Lung	4
Kidney	3
Brain	3
Lymphoma	3
Parotid gland	3
Cervix	2
Bladder	1
Bone	1
Leukemia	1
Liver	1
Multiple myeloma	1
Sarcoma	1
Stomach	1
Missing	4
Other	11
Total	188

eTable 3. Means of Detection of Breast Cancer

	**No MRI group N =103**	**MRI group N = 241**
MRI	0	106
Other screening method (any)	28	42
Ultrasound	6	14
Mammogram	22	28
Both	0	0
Palpable	59	78
Myself	57	75
Doctor	2	3
At preventive surgery	2	9
Other/unknown	14	6

eTable 4. Characteristics of Invasive Breast Cancers in the No MRI Surveillance Group and the MRI Surveillance Group

	**No MRI group**<br>**n = 79**	**MRI group**<br>**n = 205**
Age of diagnosis (years)	45.8 (32-69)	46.5 (31-72)
Year of diagnosis	2007.5 (1997-2019)	2011.4 (1999-2021)
Size (cm)	3.2 (0.3-30)	2.3 (0.1-9.0)
Missing	10	20
Node status		
Positive	23 (34.3%)	35 (19.7%)
Negative	44 (65.7%)	143 (80.3%)
Missing	12	27
ER-positive		
Positive	24 (37.5%)	66 (36.3%)
Negative	39 (60.9%)	101 (55.5%)
Borderline	1 (1.6%)	15 (8.2%)
Missing	15	23
Chemotherapy		
No	8 (12.9%)	30 (18.3%)
Yes	54 (87.1%)	134 (81.7%)
Missing	17	41
Initial Surgery		
Lumpectomy	14 (22.2%)	36 (19.7%)
Unilateral mastectomy	47 (74.6%)	136 (74.3%)
Bilateral mastectomy	2 (3.2%)	11 (6.0%)
Missing	16	22
Radiation therapy		
No	37 (61.7%)	112 (72.7%)
Yes	23 (38.3%)	42 (27.3%)
missing	19	51
Died of breast cancer (%)	17 (21.5%)	14 (6.8%)
10 -year actuarial breast cancer survival	73.1%	88.5%

© 2024 Lubinski J et al. *JAMA Oncology*

eTable 5. Characteristics of the 14 Women Who Died of Invasive Breast Cancer in the MRI Surveillance Group

Main index	Country	Age at first MRI	Year of first MRI	Year of last MRI	Year of breast cancer	Total MRI at/before dx	Age of dx	Means of diagnosis	Size (CM)	Nodes	Age at death
5279	Canada	59	2001	2005	2005	5	62	MRI	6.0	Neg	73
5934	Poland	40	2011	2013	2015	2	44	Palpable	1.3	Neg	47
6527	Norway	40	2006	2008	2008	2	41	MRI	0.5	Neg	46
6764	Poland	41	2009	2009	2010	1	42	Palpable	2.0	Neg	45
7166	USA	42	2004	2004	2004	1	42	MRI	6.0	.	43
15368	Poland	53	2008	2008	2008	1	53	MRI	0.8	Neg	59
16349	Poland	47	2008	2013	2017	4	55	MRI	1.5	Pos	57
16651	Poland	33	2007	2007	2009	1	35	Palpable	2.0	Pos	39
16734	Poland	36	2008	2008	2008	1	36	Palpable	1.6	Neg	46
18086	Poland	61	2008	2011	2014	3	66	MRI	1.7	Pos	72
18535	Poland	53	2013	2013	2013	1	53	MRI	1.5	Neg	55
19806	Canada	54	2008	2008	2011	1	56	Mammogram	6.0	Pos	59
23420	Norway	35	2006	2006	2008	1	37	Palpable	0.5	Neg	39
23559	Norway	46	2005	2005	2005	1	46	MRI	0.5	Pos	51
