# Supplementary material for: MRI Surveillance and Breast Cancer Mortality in Women With BRCA1 and BRCA2 Sequence Variations
Source: JAMA Oncol. 2024 Feb 29;10(4):493–9. doi: 10.1001/jamaoncol.2023.6944 (PMC10905376; doi:10.1001/jamaoncol.2023.6944)
Supplement: Supplement 2. — Nonauthor Collaborators [file jamaoncol-e236944-s002.pdf]

Supplemental Online Content: Nonauthor Collaborators
*First name, last name, and suffix (if applicable) are required and will appear in PubMed.

*Group Name(s): Hereditary Breast Cancer Clinical Study Group							
***First Name and Middle Initial(s)**	***Last Name**	***Suffix (eg, Jr, III)**	Academic Degrees	Institution	Location (city, state/province, country)	Role or Contribution, eg, chair, principal investigator	Group (if more than 1 Group listed in the byline) and/or Subgroup (eg, Steering Committee)
Kevin	Sweet		MSc	Ohio State University - Comprehensive Cancer Center	Colombus, Ohio, USA	data contribution	Hereditary Breast Cancer Clinical Study Group
Leigha	Senter		MSc	Ohio State University - Comprehensive Cancer Center	Colombus, Ohio, USA	Site PI	Hereditary Breast Cancer Clinical Study Group
Howard	Saal		MD	Cincinnati Children's Hospital Medical Cener	Cincinnati, OH 45229, USA	data contribution	Hereditary Breast Cancer Clinical Study Group
Lea	Velsher		MD	North York General	North York, ON, Canada	Site PI	Hereditary Breast Cancer Clinical Study Group
Susan	Armel		MSc	Princess Margaret Hospital	Toronto, ON, Canada	data contribution	Hereditary Breast Cancer Clinical Study Group
Jeanna	McCuaig		MSc	University Health Network	Toronto, ON, Canada	data contribution	Hereditary Breast Cancer Clinical Study Group
Seema	Panchal		MSc	Sinai Health Systems	Toronto, ON, Canada	data contribution	Hereditary Breast Cancer Clinical Study Group
Aletta	Poll		MSc	Women's College Hospital	Toronto, ON, Canada	data contribution	Hereditary Breast Cancer Clinical Study Group
Edmond	Lemire		MD	University of Saskachewan	Saskatoon, SK, Canada	Site PI	Hereditary Breast Cancer Clinical Study Group
Kim	Serfas		MSc	Manitoba Association of Health Care Professinals	Winnipeg, MB, Canada	Site PI	Hereditary Breast Cancer Clinical Study Group
Robert	Reilly		MD	St. Mary Medical Center.	Langhorne, Pennsylvania	data contribution	Hereditary Breast Cancer Clinical Study Group
Josephine	Costalas		MD	Thomas Jefferson University Hospitals	Morrisville, Pennsylvania	data contribution	Hereditary Breast Cancer Clinical Study Group
Stephanie	Cohen		MSW	Centre for Addiction and Mental Health	Toronto, ON, Canada	Site PI	Hereditary Breast Cancer Clinical Study Group
Joanne	Blum		MD	Baylor-Sammons Cancer Center, Texas Oncology, US Oncology	Dallas, TX, USA	data contribution	Hereditary Breast Cancer Clinical Study Group
