# Supplementary material for: MRI Surveillance and Breast Cancer Mortality in Women With BRCA1 and BRCA2 Sequence Variations
Source: JAMA Oncol. 2024 Feb 29;10(4):493–9. doi: 10.1001/jamaoncol.2023.6944 (PMC10905376; doi:10.1001/jamaoncol.2023.6944)
Supplement: Supplement 3. — Data Sharing Statement [file jamaoncol-e236944-s003.pdf]

Data Sharing Statement

Lubinski. MRI Surveillance and Breast Cancer Mortality in Women With BRCA1 and BRCA2 Sequence Variations. *JAMA Oncol*. Published February 29, 2024.
doi:10.1001/jamaoncol.2023.6944

Data
Data available: No
